# Maxillary dentigerous cyst showing squamous odontogenic tumor-like proliferation: surgical approach and literature review

**DOI:** 10.4322/acr.2021.302

**Published:** 2021-07-02

**Authors:** Camila de Oliveira Barbeiro, Roberto Henrique Barbeiro, Heitor Albergoni da Silveira, Luciana Yamamoto de Almeida, Jorge Esquiche León, Andreia Bufalino

**Affiliations:** 1 Universidade Estadual Paulista (UNESP), Faculdade de Odontologia, Medicina Oral, Departamento de Diagnóstico e Cirurgia, Araraquara, SP, Brasil; 2 Universidade de São Paulo (USP), Faculdade de Odontologia de Ribeirão Preto, Patologia Oral, Departamento de Estomatologia, Saúde Coletiva e Odontologia Legal, Ribeirão Preto, SP, Brasil

**Keywords:** Squamous odontogenic tumor, odontogenic cysts, dentigerous cyst, histology

## Abstract

Squamous odontogenic tumor (SOT) is a rare benign neoplasm of the jaw that likely arises from remnants of the dental lamina. It is a slow-growing lesion, with a radiolucent appearance in the central variant. Microscopically, SOT shows islands of squamous epithelium supported by fibrous stroma. In rare cases, squamous odontogenic tumor-like proliferation (SOT-LP) can be observed arising from odontogenic cysts (SOT-LPOC). Herein, we describe the case of a 42-year-old man who presented with discreet bleeding in the maxillary gingiva. Imaging revealed a well-defined, ovoid-shaped lesion with sclerotic margins involving tooth #18 in the intraosseous location. Fine needle aspiration supported the cystic nature of the lesion. After surgery, microscopy revealed a dentigerous cyst showing SOT-LP features. There was no recurrence after a 3-year follow-up. To the best of our knowledge, this is the first report of a dentigerous cyst showing SOT-LP features in the maxilla. Such cysts should be identified to avoid misdiagnosis, with the finding having therapeutic and prognostic implications.

## INTRODUCTION

Squamous odontogenic tumor (SOT) is a rare benign neoplasm histologically characterized by islands of well-differentiated squamous epithelium supported by a fibrous stroma.^1^^,^^2^ To date, approximately 110 cases of SOT have been reported, with the vast majority (n=102) representing central lesions that are radiographically characterized as triangular radiolucent unilocular lesions. In contrast, peripheral SOT does not show radiographic alterations, but can eventually cause loss of the underlying bone. Central SOT affects individuals with a mean age of 35 years, whereas peripheral SOT affects older patients, with a mean age of 45 years. The maxilla and mandible are equally affected by both lesions, with a low recurrence rate.^3^^,^^4^

Occasionally, squamous odontogenic tumor-like proliferation (SOT-LP) can be detected in association with odontogenic cysts (SOT-LPOC).^5^ These lesions show islands of benign squamous epithelium within the cystic capsule, most commonly in radicular cysts, followed by dentigerous cysts, and less commonly, in residual and lateral periodontal cysts.^3^^,^^6^^-^^9^ Considering the biological behavior of central SOT, which can present bone expansion and cortical perforation, the SOT-LP does not appear to influence the biological behavior of the odontogenic cyst in which it occurs.^1^^,^^3^^,^^5^ However, their identification is critical due to their therapeutic and prognostic implications. To the best of our knowledge, this is the first case of a maxillary dentigerous cyst showing SOT-LP features.

## CASE REPORT

**Figure 1:**
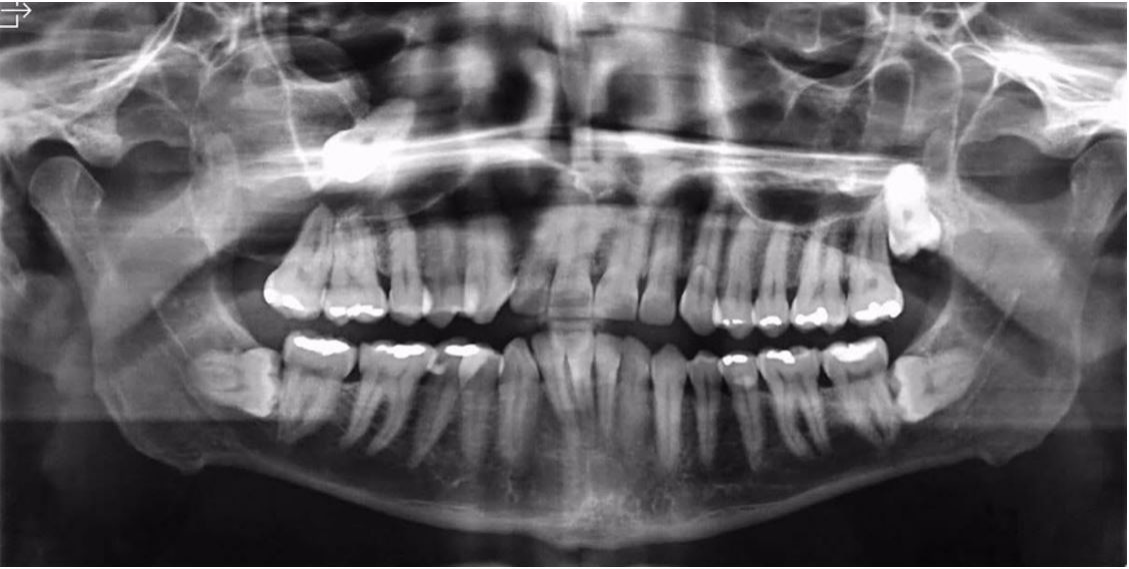
Panoramic radiography showing a unilocular radiolucent lesion associated with tooth #18 displaced into the maxillary sinus.

A 42-year-old man was referred to our service with gingival bleeding at the level of the upper right second permanent molar. According to the patient, previous periodontal treatment was performed without any clinical improvement. Intraoral examination revealed an asymptomatic, slight expansion of the buccal cortical bone at the level of the molar region. Radiographic examination revealed a well-circumscribed ovoid radiolucency around the crown of tooth #18, which was displaced into the maxillary sinus (Figure 1).

**Figure 2:**
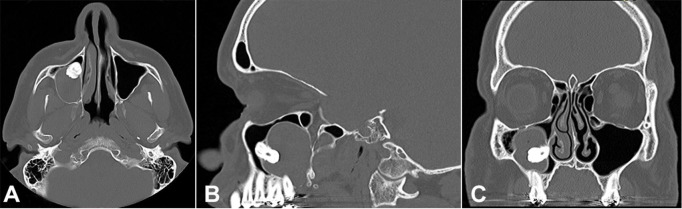
CT showing the cystic aspect of the lesion in axial (**A**), sagittal (**B**), and coronal (**C**) planes.

Computed tomography (CT) confirmed the cystic aspect of the lesion (Figure 2), and angiotomography indicated no vascular component. Fine needle aspiration supported the cystic nature of the lesion. The clinical differential diagnoses indicated it to be a dentigerous cyst, odontogenic keratocyst, and ameloblastoma.

**Figure 3:**
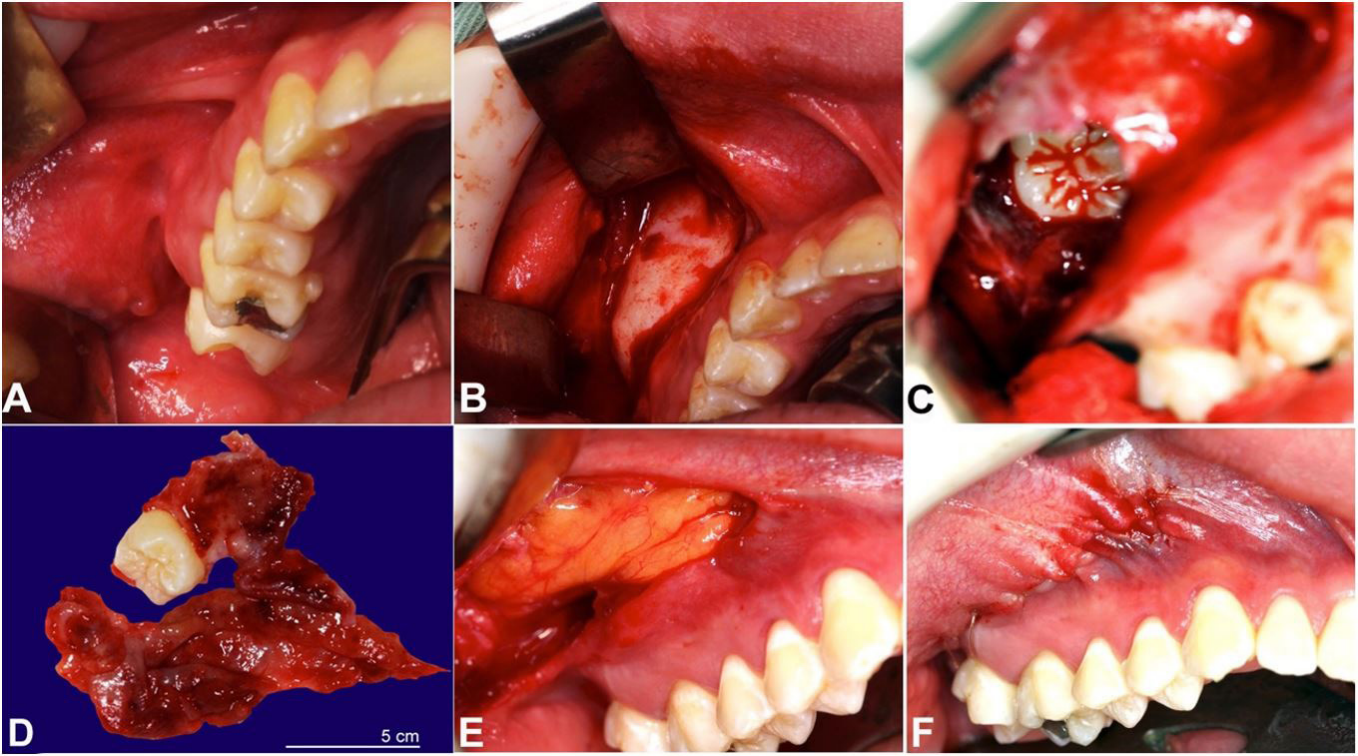
**A** – The Caldwell-Luc surgical technique; **B** – Dissection of the cyst wall; **C** – Enucleation of the lesion and visualization of the involved tooth; **D** – Macroscopic aspect of the lesion associated with tooth #18; **E** – Combined flap technique; **F** – Immediate post-operative.

The Caldwell-Luc surgical technique, which enables the enucleation of the lesion and the involved tooth, was performed under general anesthesia to access the maxillary sinus. A sublabial approach was employed through the canine fossa by making a circular incision (Figure 3A), the mucoperiosteal flap was raised, and cyst wall dissection was performed (Figure 3B), allowing the removal of the entire lesion and involved tooth (Figure 3C). The buccal fat pad was then mobilized by blunt dissection and exposed until the most appropriate volume was available for closure of the cavity. For this purpose, the combined flap technique and resorbable sutures were used (Figure 3D-F).

**Figure 4:**
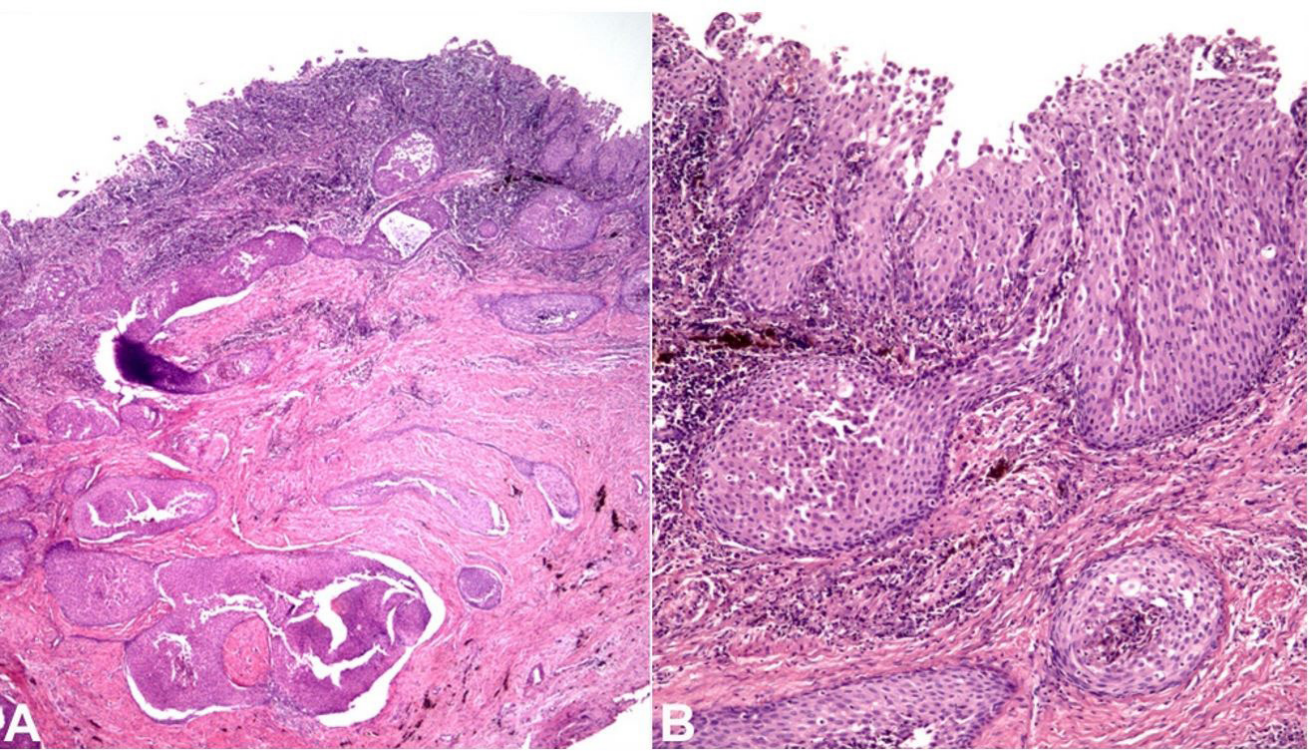
**A** – Histopathological analysis showing a cystic lesion lined by nonkeratinized, stratified squamous epithelium, containing several islands of benign-appearing squamous epithelium in the cystic capsule (H&E staining, x25); **B** – In a close-up view, an island of benign-appearing squamous epithelium arising from the epithelial lining can be observed (H&E staining, x100).

Microscopy revealed a cystic epithelium supported by a fibrous connective tissue capsule surrounded by moderate chronic inflammatory cellular infiltrate. Notably, several islands of benign-appearing squamous epithelium within the cystic capsule were observed (Figure 4A). Moreover, a focal area exhibited an island of benign-appearing squamous epithelium arising from the cyst lining epithelium (Figure 4B).

**Figure 5:**
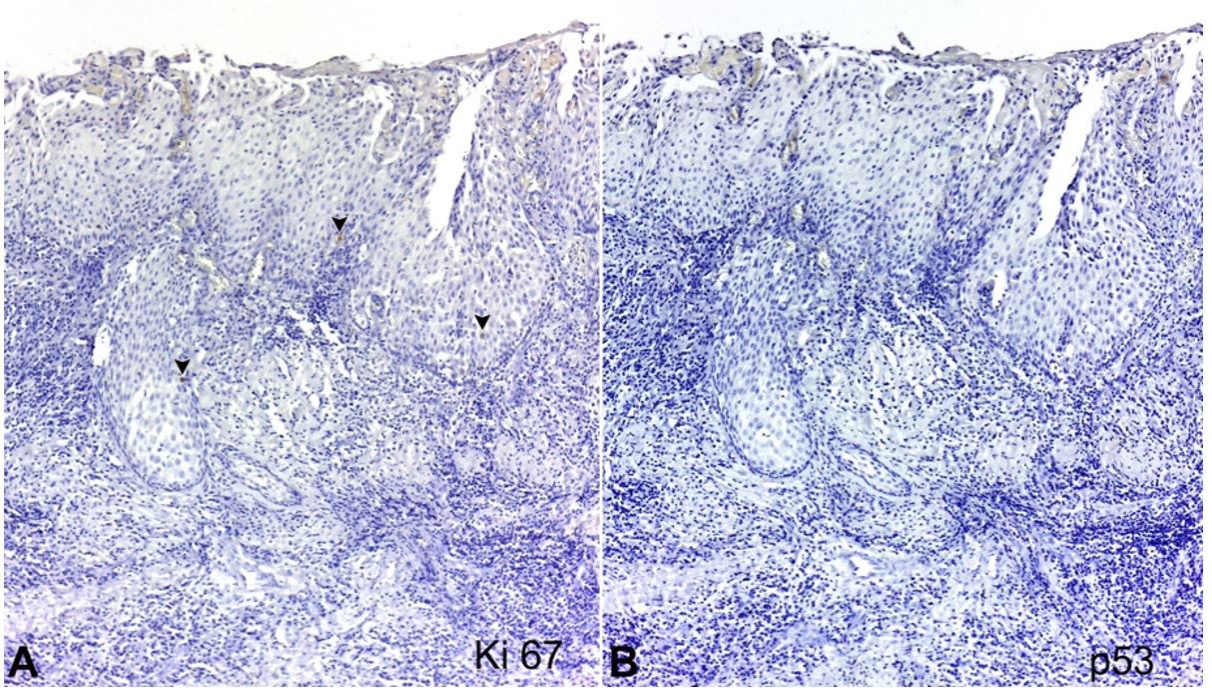
Immunohistochemical analysis. **A** – Sparse cells positive for Ki-67 (arrows); **B** – Absence of p53 expression. These findings support the benign nature of the lesion.

Immunohistochemistry indicated that only scarce epithelial cells were Ki-67 positive, whereas the lesion was negative for p53 (Figure 5A-B), supporting its benign nature. A final diagnosis of a dentigerous cyst showing SOT-LP features was made. After 3 years of follow-up, no recurrence or alteration in the lesion area was observed.

## DISCUSSION

SOT-LP was first described in 1979 by Wright,^5^ who reported four cases presenting numerous islands of benign-appearing squamous epithelium detaching from the odontogenic cyst lining epithelium. These histological features are similar to those observed in SOT, except that in SOT-LP, epithelial proliferation is confined to the cyst wall.^1^^,^^5^^-^^7^ For this reason, a careful inspection of the cystic capsule is necessary, which would allow differentiating SOT-LP from central SOT.^3^^,^^5^

To date, 60 cases of SOT-LPOC have been reported, affecting patients with a mean age of 44 years, without gender predilection. Most cases of SOT-LP have been reported to be associated with radicular cysts (n= 51), whereas only six cases were associated with dentigerous cysts.^3^^,^^8^^,^^10^^,^^11^ These lesions occurred more frequently in the maxilla than in the mandible in individuals with a mean age of 44 years, without gender predilection. The SOT-LPOC cases usually present a lack of bone expansion, cortical perforation, tooth mobility, tooth resorption, and recurrence, while several of these clinicopathological features can be detected in central SOT.^3^ All cases of SOT-LP arising from dentigerous cysts affected the mandible, while the current case is the first to involve the maxilla.^3^^,^^4^ Radiographic analysis showed a unilocular lesion associated with a displaced or unerupted tooth. The treatment of choice was enucleation, without recurrence.^3^^,^^4^ A summary of the clinicopathological features of SOT-LP arising from dentigerous cysts is shown in Table 1.

**Table 1:** Summary of the clinical characteristics of squamous odontogenic tumor-like proliferation in dentigerous cysts

	Age (y)	Site	Gender	Involved tooth	Tooth displaced/ unerupted	Symptoms	Bone resorption /expansion	Treatment	Relapse	F-up
Wright^5^	45	Md	F	2^nd^ molar	No	Yes	No	Enucleation	No	2¼y
Wright^5^	53	Md	M	3^rd^ molar	Yes	No	No	Enucleation	No	2y
Wright^5^	36	Md	M	3^rd^ molar	Yes	No	No	Enucleation	No	4m
Wright^5^	65	Md	F	3^rd^ molar	Yes	No	No	Enucleation	No	ND
Leventon et al.^6^	17	U	F	3^rd^ molar	No	No	No	Enucleation	No	7m
Fay et al.^7^	60	Md	M	2^nd^ molar	ND	Yes	No	Enucleation	Nd	2 ½y
Our case	42	MX	M	3^rd^ molar	No	No	No	Enucleation	No	3y

F= female, F-up= follow-up, m= months, M=male, Md= mandible, Mx= maxilla, ND: Not documented, U= unknown, y= years

Peripheral SOTs are extremely rare, and only eight cases have been reported, with the maxilla and mandible being equally affected.^2^^,^^12^ Women were affected more frequently than men (ratio 3:1), with a mean age of 45 years.^3^^,^^4^ Interestingly, tooth displacement in one case of peripheral SOT and six cases of SOT-LPOC have been reported.^3^ Surgery is the treatment of choice for peripheral SOT and the prognosis is good. However, one case reported recurrence after 156 months of follow-up,^2^^-^^4^^,^^12^ and close monitoring of these lesions is recommended.

It is important to emphasize that microscopically SOT-LP can mimic a neoplasm. In the current case, histopathological features suggestive of ameloblastoma, especially the acanthomatous type, such as peripheral palisading columnar cells at the basal layer, hyperchromatic nuclei showing reverse polarization, and stellate reticulum-like cells, were not observed. Moreover, the possibility of primary intraosseous squamous cell carcinoma arising from an odontogenic cyst should be carefully excluded. In fact, unlike the current case, in malignant neoplasms, the epithelial islands exhibit marked nuclear and cellular pleomorphism and hyperchromatism, atypical mitotic figures, apoptotic bodies, and altered nuclear-cytoplasmic ratio. In this context, it is necessary to apply strict diagnostic criteria to correctly differentiate odontogenic cyst presenting SOT-LP features from squamous cell carcinoma arising from an odontogenic cyst^13^^,^^14^ due to therapeutic and prognostic implications.

Interestingly, a recent study showed an unusual occurrence of a typical SOT mimicking a dentigerous cyst around tooth #47 in a 19-year-old woman. This case was also asymptomatic and was discovered after routine radiographic examination, which after microscopic analysis, did not reveal cystic areas. The case showed only multiple islands and strands of well-developed squamous epithelium within a fibrous connective tissue stroma, corroborating the final diagnosis of SOT and highlighting the importance of a strict clinicopathological correlation.^10^

In conclusion, SOT-LPOC is an uncommon histopathological finding, similar to those observed in central SOT, which is typically a solid lesion with a more aggressive biological behavior. Accordingly, careful histopathological analysis is fundamental, as SOT-LPOC can be misdiagnosed as a primary benign or malignant epithelial neoplasm. Thus, a strict clinicopathological correlation is essential to achieving a correct diagnosis with therapeutic and prognostic implications.

## References

[1] Pullon PA, Shafer WG, Elzay RP, Kerr DA, Corio RL (1975). Squamous odontogenic tumor. Report of six cases of a previously undescribed lesion. Oral Surg Oral Med Oral Pathol.

[2] Elmuradi S, Mair Y, Suresh L, DeSantis J, Neiders M, Aguirre A (2017). Multicentric squamous odontogenic tumor: a case report and review of the literature. Head Neck Pathol.

[3] Chrcanovic BR, Gomez RS (2018). Squamous odontogenic tumor and squamous odontogenic tumor-like proliferations in odontogenic cysts: an updated analysis of 170 cases reported in the literature. J Craniomaxillofac Surg.

[4] Parmar RM, Brannon RB, Fowler CB (2011). Squamous odontogenic tumor-like proliferations in radicular cysts: a clinicopathologic study of forty-two cases. J Endod.

[5] Wright JM (1979). Squamous odontogenic tumorlike proliferations in odontogenic cysts. Oral Surg Oral Med Oral Pathol.

[6] Leventon GS, Happonen RP, Newland JR (1981). Squamous odontogenic tumor. Am J Surg Pathol.

[7] Fay JT, Banner J, Rothouse L, Kolas S, Klinger BJ, Sayers RJ (1981). Squamous odontogenic tumors arising in odontogenic cysts. J Oral Med.

[8] Simon JH, Jensen JL (1985). Squamous odontogenic tumor-like proliferations in periapical cysts. J Endod.

[9] Sala-Pérez S, Marco-Molina V, Gay-Escoda C (2013). Squamous odontogenic tumor-like proliferation in a radicular cyst: a case report. J Clin Exp Dent.

[10] Laungani N, Hengen S, Nester C, Smith MH (2020). Pericoronal radiolucency surrounding an impacted mandibular molar. Oral Surg Oral Med Oral Pathol Oral Radiol.

[11] Unal T, Gomel M, Gunel O (1987). Squamous odontogenic tumor-like islands in a radicular cyst: report of a case. J Oral Maxillofac Surg.

[12] Anjana R, Murali N, Malathi N, Suresh RR (2016). Management of a rare case of peripheral squamous odontogenic tumor of the gingiva. J Indian Soc Periodontol.

[13] Swinson BD, Jerjes W, Thomas GJ (2005). Squamous cell carcinoma arising in a residual odontogenic cyst: case report. J Oral Maxillofac Surg.

[14] Oliveira JA, Costa IM, Loyola AM (2006). Squamous odontogenic tumor-like proliferations (SOT-LP) versus intraosseous squamous cell carcinoma in residual cyst. J Oral Maxillofac Surg.

